# 

**Published:** 1890-07

**Authors:** 


WHOLE NUMBER, CCCXLVIII.

New Series.

July, 1890.

VOL. XXIX. No. 12.

### BUFFALO MEDICAL AND SURGICAL JOURNAL.

Established 1845 by AUSTIN FLINT, M. D.

**Editors:**

THOS. LOTHROP, M. D. W. W. POTTER, M. D.

**Associate Editors:**

FRANK HAMILTON POTTER, M. D. JAMES WRIGHT PUTNAM, M. D.

WILLIAM C. KRAUSS, M. D. JOHN PARMENTER, M. D.

SCRIPTA FERUNT ANNOS

PUBLISHED MONTHLY.

SUBSCRIPTION PRICE, $2.00 IN ADVANCE; OR $2.50 A YEAR IF NOT PAID IN ADVANCE.

EUROPEAN AGENCY: TRÜBNER & CO., 57 AND 59 LUDGATE HILL, LONDON, E. C.

BUFFALO: 1890.

FOREIGN SUBSCRIPTION, $2.50

Entered at the Post-Office at Buffalo, N. Y., as Second-class Mail Matter.

*Times Printing House, Buffalo, N. Y.*

